# Acknowledgement to Reviewers of *Nanomaterials* in 2017

**DOI:** 10.3390/nano8010039

**Published:** 2018-01-13

**Authors:** Nanomaterials Editorial Office

**Affiliations:** MDPI AG, St. Alban-Anlage 66, 4052 Basel, Switzerland

Peer review is an essential part in the publication process, ensuring that *Nanomaterials* maintains high quality standards for its published papers. In 2017, a total of 464 papers were published in the journal. Thanks to the cooperation of our reviewers, the median time to first decision was 17.00 days and the median time to publication was 42.00 days. The editors would like to express their sincere gratitude to the following reviewers for their time and dedication in 2017:

Abdel-Mohsen, A. M.Lin, Yang-weiAbdul Samad, YarjanLin, Chih-MingAbend, MichaelLin, Chi-ChangAbou Neel, Ensanya A.Lin, Tai-YuanAbu-Reziq, RaedLin, QianqianAcha, VictorLin, GuitingAdamcakova-Dodd, AndreaLin, Hung-YinAguilera-Servin, Juan LuisLinert, WolfgangAhi, YadvinderLiopo, AntonAhmed, IftyLips, Katrin S.Ahmed, MaryaLiu, HaipengAhmed, FaridLiu, QingkunAires, AlfredoLiu, ZhixiaAkazawa, HouseiLiu, YinAlam, ParvezLiu, FeiAlarcon, EmilioLiu, XiaoxiAlbadarin, Ahmad B.Liu, SongAlbini, AngeloLiu, RenAlbo, JonathanLiu, YanbiaoAlcoutlabi, MatazLiu, ShaominAldakov, DmitryLizundia, ErlantzAlexiou, ChristophLocatelli, AndreaAlexis, FrankLojou, ElisabethAljabali, AlaaLok, Chun NamAlkaisi, MaanLolla, DineshAlonso, JavierLongoni, GianlucaAlonso, ConcepciónLópez, Moisés CanleÁlvarez, José IgnacioLópez, David OrencioAmadelli, RossanoLópez Antón, RicardoAmreddy, NarsireddyLópez-Lorente, Ángela I.Anderson, Anne J.Losurdo, MariaAndreopoulou, Aikaterini K.Lu, ChenyangAndruszkiewicz, RyszardLuan, BinquanAnsón Casaos, AlejandroLudwig, FrankAradilla, DavidLuhrs, Claudia C.Arany, PraveenLuna, CarlosArias, JavierLundstrom, KennethAriga, KatsuhikoLuong, Dzung DinhArnusch, ChristopherLyons, John G.Arora, AshishLyubchenko, YuriArpicco, SilviaMacdonald, ThomasArvapally, Ravi KumarMacucci, MassimoAsai, DaisukeMaczka, MirosławAta, SeisukeMadasamy, ThangamuthuAvnir, DavidMahmood, KhalidAw, KeanMajewski, Jacek A.Babu, AnishMajor, IanBachaga, TarekMajumder, KaustavBácskay, IldikóMakarona, E.Bae, Jong WookMalakooti, Mohammad H.Bae, ChangdeuckMalmstrom, JennyBaino, FrancescoMalucelli, GiulioBalandin, Alexander A.Manciu, FeliciaBald, IlkoMandracci, PietroBálint, ErikaMangoni, Maria LuisaBalme, SébastienManickam, PandiarajBampoulis, PantelisMannell, HannaBan, MasahitoManthina, VenkataBanach, MarcinMao, ChuanbinBandala, Erick R.Marcelli, RomoloBandodkar, Amay J.Marchesan, SilviaBandyopadhyay, SulalitMarchiol, LucaBao, YupingMarino, AttilioBao, YingMarpu, Sreekar BabuBarbillon, GrégoryMartin, James D.Baroutian, SaeidMartin, Stephen M.Barradas, ThaísMartin, IsidroBarranco, AngelMartínez, CristinaBarroso, MargaridaMartino, SabataBasu, AnirbanMartins, Rui C.Beall, Gary W.Martín-Yerga, DanielBeard, James D.Mascia, LenoBechelany, MikhaelMassiani, PascaleBégin, DominiqueMásson, MárBella, FedericoMasuda, YoshitakeBellan, Leon M.Masuo, SadahiroBelver, CarolinaMaterer, Nicholas F.Bencardino, FrancescoMatsumoto, KazuhikoBenito, José ManuelMatthias, LeinBernards, MatthewMavrantzas, VlasisBerret, Jean-FrançoisMcGuinness, Garrett B.Bertocci, FrancescoMeier, Samuel M.Bhaskaran, HarishMeinardi, FrancescoBhatnagar, AmitMelchionna, MicheleBianco, I. D.Memarzadeh, KavehBiernat, JanMéndez, BianchiBillaud, JulietteMeng, LijianBishay, PeterMeng, XinBiswas, SouvikMessori, LuigiBlach, Jean-FrançoisMetha, GregBlayo, AnneMeulenberg, ElineBloh, Jonathan Z.Michaelis, David J.Bludov, YuMichailidis, ΝikolaosBochenkov, Vladimir E.Michel, Carlos R.Boghossian, ArdemisMilanesio, MarcoBolotov, LeonidMiller, Christopher J.Bonani, WalterMills, DavidBorkowski, AndrzejMinakshi, ManickamBoudaden, JamilaMinami, IchiroBouropoulos, NikolaosMinea, Alina AdrianaBoyd, Robert D.Mirabella, SalvoBrendlé, JocelyneMiroshnichenko, AndreyBrooks, Donald E.Mishra, YogendraBrouwers, JosMisra, DeveshBrutti, SergioMita, TsuyoshiBunch, Joseph ScottMitchell, Scott G.Burlage, RobertMitchell, JohnBusquets, Maria AntòniaMitomo, HideyukiCabaleiro, DavidMitov, MichelCai, ZhengxinMitsumata, TetsuCalero, Olga CaballeroMiyaji, HirofumiCall, MindyMiyatake, TomohiroCallahan, Laura SmithMiyazaki, ToshikiCamacho, Lucy M.Modesti, MicheleCâmara, JoséMohanta, AntaryamiCamerel, FranckMohd-Yasin, FaisalCaminati, GabriellaMomeni, KasraCamões, AiresMontenegro, LuciaCampuzano Ruiz, SusanaMonti, AlessioCao, WeiMorelli, CatiaCaputo, Gregory A.Moreno Pineda, EufemioCaracciolo, GiulioMori, WasukeCarli, StefanoMoschini, ElisaCarosio, FedericoMosquera, Maria JCaruntu, DanielaMoure, AlbertoCaruntu, GabrielMoussa, SherifCasari, CarloMuench, FalkCasettari, LucaMuller, MichaelCassee, FlemmingMüller, GerhardCastaldi, GiuseppeMünch, AlexanderCastell, PereMuniz-Miranda, FrancescoCastñeiras, AlfonsoMurase, NorioCastrucci, PaolaMurcia-López, S.Casu, AlbertoMurphy, CormacCauda, ValentinaMutlu, HaticeCavaliere, SaraMuzykantov, VladimirCavallotti, PietroNadtochenko, VictorCavicchi, KevinNagaoka, HiroyukiCefalas, A. C.Nagase, YuCen, YinjieNagasubramanian, GanesanCesano, FedericoNair, RemyaCetin, Arif EnginNakajima, AnriCevenini, LucaNakamura, HiroyukiCha, Sang-HoNakashima, TakuyaChae, HeeyeopNakayama, MasaharuChandra, SubhashNalbach, PeterChang, Chih-YuNallathamby, Prakash D.Chang, Tso-Fu MarkNandwana, VikasChang, Sheng-PoNattestad, AndrewChantana, JakapanNaumov, AntonChase, George G.Naviglio, SilvioChattopadhyay, SurojitNayak, AnimeshChen, HongweiNazare, ShonaliChen, YijiaNees, MatthiasChen, Chia-YunNegishi, NobuakiChen, BiqiongNegro, EnricoChen, FanNguyen, Duc ThanhChen, JuanNguyen, Hoa HongChen, YiNielsen, Line HagnerChen, ShujianNiemirowicz, KatarzynaChen, Yi-TingNiidome, TakuroChen, ShuangqiangNishijima, YoshiakiChen, Lung-ChienNishikiori, HiromasaChen, XiNohe, AnjaChen, Szu-yuanNonappa, NonappaChen, Cheng-LungNoor, NuruzzamanChen, Pang-ShiuNoubactep, ChicgouaCheng, Yang-TseNovák, IgorCheng, Fong-YuNovotná, VladimíraCheng, XingguoNuxoll, EricCheng, ZhenzhouNyström, GustavCheng, XingguoObraztsov, AlexanderCheng, Fong-YuOchiai, TsuyoshiCheong, Yuen-KiOh, Jae-MinCheong, In WooOh, Min-WookChiang, ShirleyOh, Daniel S.Chikan, ViktorOhno, SeigoChinappi, MauroOka, ChiemiChiono, ValeriaOkochi, MinaChiu, Chi-chengOkubo, MasayoshiCho, Dae WonOlivera-Pastor, PascualCho, Sung-JinOmbres, LucianoCho, Kuk YoungOmrani, EmadChoi, SeongHoOnbasli, M. C.Choi, Myong YongOperamolla, AlessandraChoi, Soo-JinOpitz, JörgChoi, SamjinOrbea, AmaiaChoi, Jong HyunOu, JianzhenChoi, Nag JungOuyang, ZiChoi, WonjoonPablos, CristinaChoi, JoshuaPacchierotti, FrancescaChong, Lee-GauPaik, UngyuChou, Jung-ChuanPala, NezihChou, Shih-FengPalchoudhury, SoubantikaChoy, Young BinPalermo, Edmund F.Christensen, Jørn B.Pan, HengCicala, GianlucaPanah, Saeid MasudyCicciu, MarcoPane, SalvadorCiesielski, Peter N.Pantano, MariaCinti, StefanoPanzarasa, G.Čiplys, DaumantasPao, Chun-WeiClark, AlasdairPaolo, ArosioCoelho, José A.Papageorgiou, George Z.Coleman, Anthony W.Papini, EmanueleColombo, MiriamPardanaud, CédricColvin, Emily K.Park, In-KyuComparelli, R.Park, Sol-MoiConti, BicePark, Jang-UngCornell, B.Park, JonghyunCosco, DonatoPark, Kyeong-WonCozzolino, AnthonyPark, WansuCran, Marlene J.Park, Kang HyunCremar, Lee DanielParker, LindsayCroce, Anna CletaParlett, ChristopherCui, HaitaoPasini, DarioCulbertson, Christopher T.Passaglia, ElisaCzech, BożenaPassalacqua, RosalbaDacarro, GiacomoPatel, BrijeshkumarDaftarian, PirouzPato Doldan, BreoganDang, AleiPatterson, JenniferDaniel, ChristophePaul, Titan C.Darling, SethPaulmurugan, RamasamyDavidchack, Ruslan L.Paulsen, HaukeDawn, ArnabPaulusse, JosDe Aza, PiedadPeijnenburg, Willie J. G. M.De La Mata, Francisco JavierPellejero, IsmaelDe La Prida, Victor ManuelPennelli, GiovanniDe Lima, RenataPensabene, VirginiaDe Rosa, Maria CristinaPerepelyuk, MarynaDe Stefano, LucaPérez-Juste, JorgeDearden, John C.Périgo, Élio AlbertoDebliquy, MarcPerrot, ArnaudDelbecq, FredericPersano, LuanaDelehanty, JamesPeters, Ruud J. B.D'Elios, Mario MilcoPetit, TristanDeLong, Robert K.pezzella, alessandroDeng, PengPhan, Manh-HuongDenizot, BenoitPhilipose, U.Deo, RandhirPhung, Quoc TriDeria, PravasPhuoc, Nguyen N.Derosa, PedroPhuong, Nguyen-TriDhakal, AshimPiana, MicheleDhakshinamoorthy, AmarajothiPichon, ChantalDhau, JaspreetPielichowska, KingaDi Lorenzo, Maria LauraPierron, Olivier N.Di Bartolomeo, AntonioPina, SandraDi Credico, BarbaraPini, ValeriosDi Mauro, AlessandroPinto, ArturDi Tommaso, DevisPiro, BenoitDias, JulianaPirola, CarloDíaz-Cruz, José ManuelPistone, AlessandroDidar, TohidPlakas, KonstantinosDifato, FrancescoPleskova, SvetlanaDikalov, SergeyPolavarapu, LakshminarayanaDillert, RalfPolimeni, AntonellaDilonardo, ElenaPolimeno, AntoninoDimakis, NicholasPolitano, AntonioDimitratos, NikolaosPolyak, BorisDinh, ToanPons, ThomasDinku, AbayPopat, AmiraliDiroll, BenjaminPope, MichaelDobrev, DobromirPorcaro, FrancescoDomb, AviPostigo, Pablo AitorDomine, Marcelo EduardoPouponneau, PierreDonarelli, MaurizioPourrahimi, Amir MasoudDong, MingdongPrakash, AdithyaDonnarumma, GiovannaPrassl, RuthDoucet, Jean PierrePredoi, DanielaDu, XuProkopovich, PolinaDu, HenryProvine, J.Du, XushengPu, HaihuiDuboz, Jean-YvesPu, KanyiDumee, LudovicPuiggali, JordiDunnill, CharliePut, BrechtDutta, MitraPuurunen, RiikkaDyett, BrendanQuijada-Garrido, IsabelDziedzic, ArkadiuszQureshi, AnjumDziubla, ThomasRadecki, JerzyEbara, M.Raghunathan, VijayEbong, AbasifrekeRagona, lauraEdirisinghe, MohanRahier, HubertEfstathopoulos, Efstathios P.Rahman, MasudurEgawa, YuyaRahme, KamilEl-Gendy, Ahmed A.Rakotondrabe, MickyEl-Safty, Sherif A.Ramanavicius, ArunasEl-Seedi, Hesham R.Ramasamy, MohankandhasamyEmoto, MakotoRamirez-Vick, JaimeEndres, FrankRana, DipakEngelke, HannaRandall, ChristopherEpifani, MauroRankin, StephenErdem, EmreRaposo, MariaEreña, JavierRaptis, IoannisEsposito, Emilio XavierRatovitski, EdwardEsposito, GennaroRavelli, DavideEstellé, PatriceRazavi, PedramEstevez, M. CarmenReehal, H. S.Estrin, YuriRemediakis, IoannisEtacheri, VinodkumarRey, AlejandroEvangelisti, ClaudioRhéaume, ÉricEvans, BenjaminRicco, RaffaeleFacsko, StefanRider, A. N.Fakhrullin, Rawil F.Ridi, FrancescaFalconi, ChristianRighi, Maria CleliaFalmbigl, MatthiasRim, You SeungFampiou, IoannaRimoldi, LucaFang, Jia-YouRimondini, LiaFaraldos, MarisolRinaldi-Montes, NataliaFarhat, SamirRissanou, Anastassia N.Fathieh, FarhadRitala, MikkoFatyeyeva, KaterynaRivas Murias, BeatrizFaust, JamesRizza, GiancarloFeng, XundaRizzi, Gian AndreaFeng, Z. VivianRizzolio, FlavioFernandes, EduardaRocchitta, GaiaFernández Romero, Juan ManuelRoddaro, StefanoFerrari, MaurizioRodriguez, NoelFerré-Borrull, JosepRodríguez, Teresa PinedaFettkenhauer, ChristianRodriguez Fortuno, FranciscoFiorito, SilvanaRoger, LeblancFischer, DagmarRogl, GerdaFlores, AraceliRondeau-Gagne, SimonFollain, NadègeRoppolo, IgnazioForgan, RossRossignol, JulienForment-Aliaga, AliciaRoth, Stephan V.Fortenberry, RyanRtimi, SamiFrancesco, Fabio DiRubenstein, David A.Francia, CarlottaRubis, BlazejFrey, MargaretRuffino, FrancescoFrisenda, RiccardoRuiz, M. PilarFröhlich, EleonoreRuman, TomaszFromm, KatharinaRumyantseva, MarinaFrosini, MariaRurali, RiccardoFu, Yen-peiRusciano, GiuliaFu, YongzhuRusnati, MarcoFu, LiRusso, MariaFujita, Shin-ichiroRyan, James G.Fukuda, TakeshiRyan, David K.Gabrielson, Nathan P.Ryu, Du YeolGaletti, MariclaSa, JacintoGállego, IsaacSaafi, MohamedGao, GuojunSabate, RaimonGao, TongchuanSacco, AdrianoGarbovskiy, YuriySadowska, BeataGarcia, Coralia V.Safiuddin, Md.García, TaniaSagan, SandrineGarcía-Armada, PilarSagawa, TakashiGard, PaulSahu, Saura C.Garrido-Cardenas, Jose AntonioSai, HiroakiGartia, ManasSaito, MasatoGary-Bobo, MagaliSaito, KazuyaGaspar, VítorSakaebe, HikariGeissler, DanielSakai, ToshioGenchi, GiadaSakilam, SatishGeng, DonglingSalerno, AurelioGentile, PiergiorgioSamperi, FilippoGenty, EricSánchez, Miguel Ángel SogorbGeso, MoshiSánchez-García, DavidGhamsari, Behnood G.Sanli, AbdulkadirGhidelli, MatteoSannicolò, FrancescoGiacca, MauroSansone, FrancescoGiancane, GabrieleSantamaria, RitaGiouroudi, IoannaSantambrogio, PaoloGirousi, StellaSantos, AbelGiubileo, FilippoSantos, Fábio AndréGlobus, TatianaSaoud, KhaledGloria, AntonioSarker, DipakGodinho, MariaSarti, ElenaGojo, SatoshiSaruhan, BilgeGok, OzgulSasaki, MasahikoGoldoni, MatteoSassoni, EnricoGomez-Florit, ManuelSastre-Santos, AngelaGómez-Graña, SergioSato, KimiyasuGomez-Tejedor, J. A.Satriano, CristinaGonzález, SergioScala, AngelaGonzalez-Bejar, MariaSchachner, JörgGonzález-Rodríguez, María-LuisaScheeline, AlexanderGonzalez-Silveira, MartaSchmid, UlrichGopalakrishnan, GopanSchueneman, GregGopiraman, MayakrishnanSchulz, BurkhardtGordiichuk, PavloSchulz, UlrikeGoreham, ReneeSchwartzkopf, MatthiasGorrasi, GiulianaScirè, SalvatoreGotor-Fernández, VicenteScrimin, PaoloGouany, FradySeal, SudiptaGovind Rao, VishalSebastián, DavidGranet, RobertSegawa, HiroyoGranitzer, PetraSegura, José LuisGrasley, Zachary C.Seib, F. PhilippGrecov, DanaSelyanchyn, RomanGreenberger, Joel S.Senyuk, BohdanGregor, IngoSeo, HyunwoongGrivel, Jean-ClaudeSeo, Young-SooGrossi, MarcoSeo, YongbeomGrunwald, RüdigerSequeira, CésarGruselle, MichelSerrà, AlbertGrzelczak, MarekSeverino, BeatriceGspann, ThuridShaha, Sugrib KumarGuarrotxena, NekaneShahjamali, Mohammad MehdiGuella, GrazianoShapter, Joe G.Gueorguiev, G. K.Sharifi, TivaGuiot, CaterinaSharma, AnuragGulgunje, PrabhakarShaygan, MehrdadGunawidjaja, RaySheremet, MikhailGuo, XiaoqingShimizu, KazuoGuo, ZhanhuShin, HyunjungGupta, SanjuShin, S. R.Gutruf, PhilippShin, Weon GyuHa, Kyoung-SuShin, Seung WookHadjadj, AomarShu, Yi-ChungHadjiargyrou, MichaelSikora, PawelHadjikakou, Sotiris K.Siligardi, GiulianoHagita, KatsumiSilina, YuliyaHahn, Jae RyangSilva, António SantosHakonen, AronSilva, Francisco José Gomes DaHallinan Jr., DanielSilva, FilomenaHamdana, GerrySimka, WojciechHampp, NorbertSimon-Yarza, TeresaHan, Dong WookSimserides, ConstantinosHanaor, DorianŠindelář, VladimírHaniu, HisaoSingh, VijayHao, LiSingh, RanjanHao, JinsongSinha Ray, SumanHara, KenjiSirvio, JuhoHargrove, DerekSlobodian, PetrHaro, MartaSlosarczyk, AgnieszkaHashizume, MineoSmani, YounesHashmi, A. Stephen K.Smått, Jan-HenrikHayakawa, TohruSmith, Tim A. D.He, DelongSnegur, LubovHelsch, G.Snyder, JoshuaHempel, UteSobolev, KonstantinHenniges, UteSoejima, TetsuroHernando, BlancaSong, JunHerrera-Melián, José AlbertoSong, Young MinHerynek, VítSorrentino, AndreaHeuken, MichaelSoundappan, ThiagarajanHeys, JeffreySpanhel, LubomirHickey, Stephen G.Spiesz, PrzemekHiguchi, EijiSrivastava, GyaneshwarHildebrandt, ThibaudSrivastava, SamanvayaHill, JosephineStamatas, GeorgiosHinderliter, BrianStavrakis, StavrosHippler, RainerStefanidou, MariaHiramatsu, MineoStephan, HolgerHirayama, FumitoshiStobiecka, MagdalenaHirtz, MichaelStrankowski, MichałHirva, PipsaStraumal, Boris B.Hisaki, IchiroStrelniker, Yakov M.Ho, Wen-JengStride, John ArronHoffmann-Eifert, SusanneStrunk, JenniferHolade, YaoviStylianakis, Minas M.Homaeigohar, ShahinSubramania, Ganapathi S.Horvath, DragosSubramaniam, PrasadHsueh, Yi-HuangSugawa, KosukeHu, XiaoSullivan, AliceHuang, HaitaoSumboja, AfriyantiHuang, Chih-ChingSun, XiaoleiHuang, Huang-ChiaoSun, ShuhuiHuang, QijinSun, Chia-LiangHuang, Genin GarySun, Shih-JyeHuh, Yong-MinSun, LuyiHuh, Yun SukSun, ZiqiHung, Yew MunSun, MengHur, JaehyunSun, WujinHussain, BabarSunde, SveinHwang, Jun-DarSwain, Bhabani SankarHwang, Suk-WonSzeghalmi, AdrianaHynynen, KullervoSzekely, GyorgyIablokov, ViacheslavSzepvolgyi, JanosIatrou, HermisTabata, MakotoIbrahim, ToniTaglietti, AngeloIftekhar, SidraTaira, ShuIjiro, KuniharuTakada, TomoyaIncarnato, LoredanaTakayuki, BanIngebrandt, SvenTakeguchi, TatsuyaInoue, HiroshiTakeoka, ShinjiInsausti, MaiteTakoudis, Christos G.Ioannides, TheophilosTam, FeliciaIonescu, Elena RodicaTamanoi, FuyuhikoIonita, PetreTanaka, MasatoIrannejad, MehrdadTananaiko, OksanaIreland, TerryTang, Juntao (Reynard)Isa, LucioTang, BinIshikawa, RyoTaniguchi, MasateruIslam, KamrulTanner, ElizabethIsoda, KatsuhiroTaratula, OlenaIto, TaichiTarn, Mark D.Itoh, TamitakeTasca, FedericoIvanov, Ilia N.Tatullo, MarcoIvanov, IvanTayade, Rajesh J.Ivask, AngelaTempesta, MariaJacak, JarosławTeran, Francisco J.Jacobsen, Elisabeth EgholmTerry, IanJadwicienczak, WojciechTeymourian, HazhirJang, Ho WonThakur, Vijay KumarJasinski, Jacek B.Thamaraparambil Jayaram, DhanyaJassby, DavidThomas, DittrichJayant, Rahul DevTieu, Anh KietJayasinghe, Suwan N.Tilley, RichardJensen, Brian D.Ting, Richard C. H.Ji, LiTittarelli, FrancescaJi, HaoTiwari, SanjayJiang, XuchuanToca-Herrera, José LuisJiang, KeTodorova, NadiaJiang, JunhuaTojo, ConchaJimenez, Ana EvaTomalia, DonaldJin, JiefuTomic, StankoJiu, JintingTomitaka, AsahiJoe, DanielTorigoe, KanjiroJohn, Vijay T.Torres Lagares, DanielJohnston, Karen E.Toshihiko, FujimoriJones, DavidTosi, GiovanniJones, J CliffTosoni, SergioJotshi, ChandTownley, Helen E.Julien, ChristianTrandafirescu, CristinaJun, Bong-HyunTriantafillou, ThanasisJung, DaewoongTrifol, JonJung, Jong HwaTrindade, TitoJung, Ho-SupTripathi, Jitendra KumarKadimisetty, KarteekTrivedi, Evan R.Kahlert, HeikeTrung, Tran QuangKaittanis, Charalambos (Bambos)Truong Phuoc, NghiaKakarla, Raghava ReddyTsay, Chien-YieKakkar, AshokTschierske, CarstenKako, TetsuyaTsitsilianis, ConstantinosKamali, SaeedTsonos, C.Kamegawa, TakashiTsutsui, MakusuKamzin, S. AlexanderTsuyumoto, IsaoKanaev, A.Tulliani, Jean-MarcKang, Jun-GillTura, AndreaKang, Y. C.Turley, Eva A.Kang, InpilUbertini, FilippoKanik, MehmetUccelletti, DanielaKantartzis, Nikolaos V.Uddin, AshrafKartopu, GirayUjihara, MasakiKatayama-Yoshida, HiroshiUzunoğlu, AytekinKawasaki, HideyaVago, RiccardoKazakov, SergeyVaiano, VincenzoKechrakos, DimitrisValente, Manuel de AlmeidaKellesarian, SergioValladares, Luis De Los SantosKemell, MariannaVallés, CristinaKennedy, John V.Valov, IliaKevadiya, BhaveahVan Roosbroeck, KatrienKhabashesku, ValeryVan Thor, Jasper J.Khan, AtifVanEpps, J. ScottKharlampieva, EugeniaVankayala, RavirajKichambare, PadmakarVasicek, OndrejKidoaki, SatoruVenditti, IoleKim, Hak-YongVenkatesan, JayachandranKim, Young JoVenkatesh, A. G.Kim, Young-KwanVentura, Joao OliveiraKim, Young DokVerbruggen, Sammy W.Kim, Joo HyunVerma, Mohit S.Kim, Jun-HyunVernardou, DimitraKim, Gil-HoVezzu, DileepKim, Il TaeVicenzo, AntonelloKim, JeongyongVigolo, BrigitteKim, Dong-eogVilanova, XavierKim, BohyungVilla, AlbertoKim, Hak YongVinardell, MariaKim, KikangViñas, MiguelKim, Hyun JaeViñes, FrancescKim, JaheonVinkovic Vrcek, IvanaKim, Young-KwanVittorio, OrazioKim, JaehwanVolker, KörstgensKim, HojoongVrana, Nihal EnginKim, Myeong OkVriesekoop, FrankKim, Dong-HyunVyskočil, VlastimilKishimura, AkihiroWachs, IsraelKiss, JánosWahab, M. FarooqKita, EijiWaite, Matthew M.Kitajou, AyukoWakihara, ToruKityk, IwanWang, XizuKlajnert-Maculewicz, BarbaraWang, XiaoyongKlein, Lisa C.Wang, Yi-ShengKlotzkin, DavidWang, QunKnetsch, Menno L.W.Wang, ShengnianKnipp, DietmarWang, YulingKobayashi, ShunsukeWang, DiKodera, YasuhiroWang, Peng-Yuan (George)Kohtani, ShigeruWang, LuminKoinkar, PankajWang, YanKojima, ToshikatsuWang, ZhenjiaKoksharova, OlgaWang, YongmeiKolasinski, Kurt W.Wang, JeffreyKollu, PratapWang, HaidongKomasa, SatoshiWang, HuaiminKonarova, MuxinaWang, Su HeKontziampasis, DimitriosWang, XianqiaoKoole, Leo H.Watanabe, MasatoshiKoos, Antal A.Watanabe, HirobumiKopel, PavelWatras, AdamKordesch, Martin E.Weare, Walter W.Kortaberria, GalderWei, GangKoskinen, PekkaWeimer, JeffreyKotobuki, MasashiWen, Amy M.Kourkoumelis, NikolaosWeng, QunhongKoutsogeorgis, DemosthenesWeng, LihuiKovalenko, SergeyWertz, Philip W.Kowalska, EwaWhite, MarkKrantz, ClaudeWiczk, WiesławKreupl, FranzWiglusz, Rafał J.Krishnan, VenkatakrishnanWilhelm, StefanKromka, AlexanderWiniczenko, RadosławKuang, HuihuiWittmann, FolkerKubař, TomasWong, Lai MunKubo, TakanoriWong, Ming-ShowKudelski, AndrzejWu, Yu-ChunKühne, Alexander J. C.Wu, Ren-JangKukli, KaupoWu, TaoKumar, ShaileshWu, Sheng YunKumar, VivekWu, JudyKundu, JoydipWu, Shin-TsonKuo, Dong-HauWu, Ming-ChungKurlyandskaya, Galina V.Wu, Kevin C.-W.Kustov, LeonidWu, Shin-TsonKuttner, ChristianXi, KaiKuznetsov, Maxim L.Xi, WeixianKvítek, LiborXia, TianKwon, Young-WanXiao, XudongKyzioł, AgnieszkaXie, JianpingLa Deda, MassimoYamaguchi, TakumiLabib, MahmoudYamakov, Vesselin I.Lachman-Senesh, NoaYamamoto, AkiraLad, Robert. J.Yamauchi, NorikoLagrow, Alec P.Yanagida, SayakaLakshminarayana, PolavarapuYang, Kap SeungLamprou, DimitriosYang, ShoufengLaromaine Sagué, AnnaYang, Jen-ChangLarosa, ClaudioYang, DongLattuada, MarcoYang, Chih-ChinLaurenti, MarcoYao, JianLaurenzana, AnnaYates, Matthew Z.Laurila, TomiYe, EnyiLavrentovich, Oleg D.Ye, DelaiLazzara, GiuseppeYeh, Yin-TingLe, DuyYilmaz, ErolLebon, FrédéricYoshimoto, MakotoLee, Jea UkYounis, AdnanLee, SunwooYu, Hak KiLee, WooyoungYu, MengxiaoLee, Chia-hungYu, Cheng-JuLee, Sang KyuYu, ChengzhongLee, Min HyungYu, TaekyungLee, Hon ManYu, MeihuaLee, Kyoung G.Yuan, Pao-chiangLee, HosunYuba, EijiLee, HangilYue, YuanzhengLee, Kew-HoYun, Soon-IlLee, YuehchienZaharieva, IvelinaLein, MatthiasZecca, LuigiLemma, Solomon MengistuZhan, HaifeiLeong, ThomasZhang, LaichangLeporatti, StefanoZhang, XueqingLesiak, PiotrZhang, BingboLeung, IvanhoeZhang, ShaolinLevesque, Pierre L.Zhang, JingtuoLevi, AlessandroZhang, QiangLewandowski, WiktorZhang, KaiLewis, RichardZhang, JingjingLewis, DavidZhang, GuigangLhuillier, EmmanuelZhang, DayiLi, JiantongZhang, HongbinLi, XianchanZhao, Julia XiaojunLi, JinghaoZhao, Jiangbo (Tim)Li, YuanshiZhao, ZhenghangLi, TaoZheng, FanLi, ZengZhong, Yu LinLi, WeiyangZhou, JiajingLi, Po-HsienZhou, WenchaoLi, MingxingZhou, YouLi, XiaodongZhu, WeiLi, JiashenZhu, ZhigangLiberti, MicaelaZiegler-Borowska, MartaLikodimos, VlassiosZografopoulos, DimitriosLim, Soo-JeongZolotoukhina, TatianaLin, ChunRongZoppellaro, GiorgioLin, Jong-LiangZyła, GawełAlaLin, Yow-Jon


